# The Need to Distinguish Colloidal and Thermal Stability for Proteins

**DOI:** 10.1021/acs.nanolett.6c03345

**Published:** 2026-09-01

**Authors:** Cécilia Siri, Ekaterina Poliukhina, Quy Khac Ong, Francesco Stellacci

**Affiliations:** † Institute of Materials, 27218 Ecole Polytechnique Fédérale de Lausanne (EPFL), 1015 Lausanne, Switzerland; ‡ Institute of Bioengineering, Ecole Polytechnique Fédérale de Lausanne (EPFL), 1015 Lausanne, Switzerland

**Keywords:** protein thermal stability, colloidal stability, formulations, excipients, second virial coefficient, protein misfolding

## Abstract

Protein stability is crucial in the development of drug formulations and in understanding many aspects of protein behavior. It is known that both thermal and colloidal protein stability are modulated by the presence of small molecules. Because thermal stability is easier to assess than colloidal stability, it is often used as a proxy for evaluating the latter. In this Mini-Review, we argue that these two aspects of stability have to be considered separately, although they should be related in some complex way.

## Graphical abstract

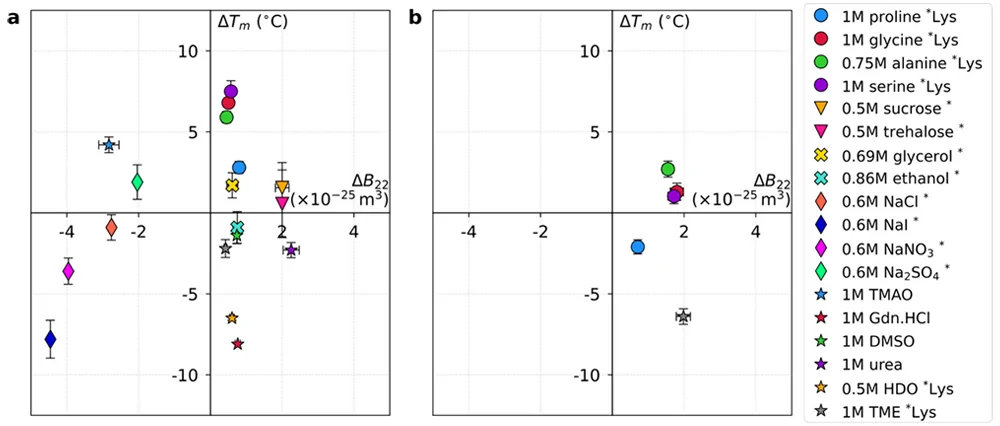

Protein stability is of central importance in biotechnology and pharmaceutical formulations.^1,2^ Significant efforts have been devoted to finding approaches that improve it. A parallel goal has been to develop rational frameworks to identify the factors that govern it.^1,3,4^

Despite its importance, the term *protein stability* is often used in a broad and sometimes ambiguous manner: stability against heating, cooling,^5^ oxidation,^6^ proteolytic or chemical degradation,^7^ and colloidal stability.^8^ It should be clearly stated that they are all distinct properties of proteins, although they all lead to loss of protein biological functions. In particular, thermal stability and colloidal stability are frequently discussed under the same umbrella.^9,10^ *Thermal stability* refers to the ability of a protein to preserve its native folded structure upon heating. It is commonly characterized by the melting temperature *T*_*m*_, defined as the midpoint of the thermal unfolding transition.^11^ *Colloidal stability*, in contrast, describes the tendency of proteins to remain homogeneously dispersed in solution without undergoing self-association, aggregation, phase separation, or other association processes over time.^9,10^ As better explained below, colloidal stability is typically measured through the second virial coefficient, *B*_22_. In simple words, the two stabilities arise from different physical interactions. Thermal stability depends mainly on intramolecular interactions between residues that stabilize the folded structure, whereas colloidal stability is governed primarily by intermolecular protein–protein interactions. For simplicity, in this Mini-Review we restrict ourselves to studying proteins in their native folded states. We therefore do not consider oligomerization pathways involving unfolded or partially unfolded species, such as those leading to amyloid formation.^12^

Both thermal and colloidal stability are highly relevant to practical applications. Protein unfolding, aggregation, and self-association can compromise bioactivity, reduce shelf life, and potentially affect patient safety.^1,13^ These forms of instability are often modulated by the composition of the solution. In protein therapeutics, small molecule (**SM**) additives – amino acids, their derivatives, sugars, polyols, buffer species – used for this purpose are commonly referred to as excipients,^4^ and their ensemble is often referred to as formulation.

In this Mini-Review, we will show that the effects of SMs on thermal and colloidal stabilities should be treated separately. Because the two properties arise from different physical interactions, an additive may affect them independently or even in opposite directions. For instance, an additive that increases a protein’s resistance to thermal unfolding does not necessarily modulate interactions between folded protein molecules in solution so as to render them more net repulsive. Conversely, an additive that improves colloidal stability by weakening protein–protein attraction does not necessarily stabilize the folded structure. This distinction is important because thermal parameters, such as *T*_*m*_, are easily measurable and widely used in early formulation screening^10,14^ as an implicit means to predict colloidal stability. If interpreted too broadly, *T*_*m*_ may be taken as an indicator of overall protein stability, including colloidal behavior. This might be misleading, as was demonstrated, for instance, by studies of therapeutic antibodies, which showed that thermal unfolding parameters alone are insufficient to predict long-term aggregation or formulation stability.^15,16^ More recent works have further emphasized that *T*_*m*_ can be a poor predictor of aggregation under storage conditions, particularly when the unfolding transition occurs well above the storage temperature.^14–16^ Here, we will illustrate that there is no direct correlation between the effect of SM on *T*_*m*_ and on *B*_22_. What we are studying, via *B*_22_ measurements, is the colloidal stability of the folded native state. Long-term colloidal stability of proteins, measured over months, is another protein property that is out of the scope of this Mini-Review. We believe that thermal and colloidal stability, while somewhat related, are nonequivalent properties of protein solutions that have to be assessed independently.

## Protein Thermal Stability and Approaches to Measure It

As temperature rises, folded proteins may lose their organized structure and undergo thermal unfolding, also known as denaturation.^17^ The higher the temperature required to induce this loss of native folded structure, the greater the protein’s thermal stability.

In the simplest two-state description, thermal unfolding is treated as a transition between the native folded state (*N*) and the denatured, or unfolded, state (*D*):

N→D

As introduced above, a common quantitative measure of thermal stability is the melting temperature, *T*_*m*_. At this temperature, the folded and unfolded populations are equal, such that [*N*] = [*D*] and *K*_*eq*_ = 1.^17^ Therefore:

$$\Delta G\left(T_{m}\right)=0$$

and, for a two-state unfolding transition:

$$T_{m}=\Delta H_{m}/\Delta S_{m}$$

where *ΔH*_*m*_ and *ΔS*_*m*_ are the enthalpy and entropy of unfolding, respectively, evaluated at *T*_*m*_. Higher *T*_*m*_ values generally indicate higher thermal stability.

The melting temperature of proteins can be measured using different techniques. One of the most common is microdifferential scanning calorimetry (**μ-DSC**).^11,18^ Its principle is simple: it measures heat-flow differences between a protein sample and a reference as the temperature changes, thereby detecting thermal events such as protein unfolding with high sensitivity.^18^ μ-DSC provides direct thermodynamic information, but it is not a high-throughput method. This can be a limitation when investigating large numbers of protein formulations.

Another method that has recently been used more widely to assess protein formulations is the thermal shift assay (**TSA**) based on protein intrinsic fluorescence.^19^ Its principle relies on measuring protein fluorescence as a function of increasing temperature. As the protein unfolds, the fluorescence intensity changes and, in many cases, the emission maximum shifts to longer wavelengths.^19^ Plotting the ratio of fluorescence intensities at 350 and 330 nm as a function of temperature allows *T*_*m*_ to be determined as the midpoint of the unfolding transition.

Another variant of TSA uses the fluorescence of an external dye, such as SYPRO Orange, instead of the protein’s intrinsic fluorescence. In this variant, the dye exhibits low fluorescence in the solvent but becomes more fluorescent upon binding to hydrophobic regions exposed during protein unfolding.^20^ The fluorescence signal therefore increases as the protein unfolds and hydrophobic patches become accessible. As in the intrinsic-fluorescence variant, *T*_*m*_ can be determined from the transition midpoint, typically by taking the first derivative of the fluorescence signal with respect to temperature.

Finally, circular dichroism (**CD**) can also be used to determine *T*_*m*_.^21^ This method relies on monitoring the loss of protein secondary structure, such as α-helices and β-sheets, as a function of temperature. As a protein unfolds, its secondary-structure content decreases, leading to changes in the CD signal.^21^ By plotting the ellipticity at a selected wavelength as a function of temperature, the unfolding transition can be followed, and *T*_*m*_ can be extracted from the midpoint of this transition.

## Protein Colloidal Stability and Approaches to Measure It

Colloidal stability is governed by the balance between repulsive and attractive protein–protein interactions (**PPIs**). Repulsive contributions may arise from electrostatic, steric, and hydration effects, whereas attractive contributions may include van der Waals, hydrophobic, depletion, and other short-range interactions.^1,3^ This balance determines whether folded proteins remain dispersed in solution or undergo self-association, aggregation, crystallization, or liquid–liquid phase separation (**LLPS**).^3,22^

Net PPIs can be described by the protein pair interaction potential, *U*(*r*), where *r* is the distance between the centroids of two protein molecules. A related thermodynamic quantity is the second osmotic virial coefficient, *B*_22_:

$$B_{22}=-2\pi \int_{0}^{\infty }\left(e^{-U\left(r\right)/k_{B}T}-1\right)r^{2}dr$$

*B*_22_ can be separated into a hard-sphere contribution and an interaction contribution:

$$B_{22}=B_{22,HS}+B_{22,int}$$

This decomposition provides a useful physical interpretation. *B*_22_ has units of volume, and is a two-body quantity that characterizes the effective interaction volume arising from pairwise interactions between protein molecules in solution. For noninteracting hard spheres, *B*_*22,HS*_ reflects the excluded volume due to the protein’s finite size. Additional interactions modify this effective volume through *B*_22,*int*_: repulsive interactions increase *B*_22_, whereas attractive interactions decrease it. Thus, positive deviations from the hard-sphere value indicate net repulsive interactions, while negative deviations indicate net attraction.^22^

Several approaches can be used to determine *B*_22_ or closely related interaction parameters (*G*_22_,^23^ *A*_2_ ^24^). For *B*_22_, a common technique is sedimentation equilibrium analytical ultracentrifugation (**AUC-SE**). This method allows for measuring the concentration profile once the equilibrium between sedimentation and back-diffusion is reached.^25^ Neglecting the higher-order many-body interactions, one can write the osmotic virial expansion in simplified form as

$$\frac{\Pi }{\rho k_{B}T}=1+B_{22}\rho$$

where Π is the osmotic pressure, ρ is the protein number density, *k*_*B*_ is the Boltzmann constant, and *T* is the temperature.^25^

Static light scattering (**SLS**) provides another common route. In SLS, the concentration dependence of the scattered intensity is analyzed using the Zimm light-scattering relation, allowing for the determination of the protein’s molecular weight and the second virial coefficient (*A*_2_, generally expressed in mol.mL.g^–2^).^9,26^ Self-interaction chromatography (**SIC**) estimates protein self-interactions from the retention of a protein on a stationary phase carrying immobilized molecules of the same protein.^27^ Osmometry is conceptually direct, as it measures osmotic pressure as a function of protein concentration and fits the result using the osmotic virial expansion.^28^

More recently, cryo-electron tomography has enabled a real-space route for measuring protein interactions.^23^ In this approach, protein solutions are vitrified, imaged in three dimensions, and individual protein positions are extracted from tomograms. From these coordinates, the radial distribution function, *g*(*r*), and the potential of mean force can be calculated. It has been shown that, for proteins, the pair interaction potential *U*(*r*) can be obtained. This allows the Kirkwood-Buff integral (*G*_22_) to be estimated from experimentally measured spatial distributions while preserving information about the interaction potential’s range and shape.

*B*_22_ must be interpreted carefully. Wills, Scott, and Winzor emphasized that apparent virial coefficients obtained from different techniques are not always equivalent.^28^ This distinction is especially important in multicomponent solutions containing salts, buffers, or excipients, where protein–SM interactions, hydrodynamic effects, and method-specific assumptions can contribute to the measured nonideality.^28^

Because PPIs influence protein phase behavior, colloidal stability can also be assessed by monitoring phase transformations, including solubility changes, crystallization, and LLPS.^3,22^ This approach is direct because it follows the phase transition of interest. However, it can require large amounts of protein and may be limited by the need to reach experimentally accessible phase boundaries. There have been attempts to relate the critical temperature *T*_*c*_ in an LLPS phase diagram with *B*_22_.^29,30^ In this paper, we focus on *B*_22_ as a compact, physically interpretable descriptor of native-state colloidal stability.

## Modulation of *T*_*m*_ and *B*_22_ by SMs

Modulation of protein thermal and colloidal stability by SMs can be tracked by changes in *T*_*m*_ and *B*_22_, respectively. In this context, stabilization is reflected by an increase in *T*_*m*_ for thermal stability (*ΔT*_*m*_ > 0) and by an increase in *B*_22_ for colloidal stability (*ΔB*_22_ > 0). Conversely, a decrease in *T*_*m*_ (*ΔT*_*m*_ < 0) indicates thermal destabilization, while a decrease in *B*_22_ (*ΔB*_22_ < 0) indicates a shift toward less repulsive, or more attractive, PPIs.

The effect of SMs on thermal stability has been studied for several decades. Compatible osmolytes such as glycine betaine and sarcosine were shown to increase the thermal stability of RNase A and lysozyme at high molar concentrations while preserving enzymatic activity.^31^ Amino acids can also increase protein thermal stability, although the effect depends on both the amino acid and the protein considered.^32^ In monoclonal antibody formulations, basic amino acids have been reported to improve thermal stability during purification and formulation.^33^ Sugars can also act as strong thermal stabilizers; for example, trehalose increased the *T*_*m*_ of RNase A by up to 18 °C at a concentration of 2 M.^34^ In contrast, some amphiphilic SMs can destabilize folded proteins, as shown for alkanediols using lysozyme as a model globular protein.^35^

SMs also modulate the native-state colloidal stability of proteins^8^ and interactions between nanoscale systems.^36^ Several free amino acids, including glycine, proline, arginine, and glutamine, were shown to increase *B*_22_ for protein,^8^ with detectable effects even at millimolar concentrations.^37^ Amino acid derivatives and mixtures can further tune PPIs in an additive manner.^38^ These effects extend to phase behavior: amino acids modulate LLPS *in vitro* and *in vivo*,^8,39^ while taurine suppresses LLPS of lysozyme and lysozyme–ovalbumin mixtures.^40^

Together, these studies show that SMs can affect both *T*_*m*_ and *B*_22_. However, the two responses should not be assumed to be linked in a simple manner. Therefore, to illustrate this point, we provide here a direct comparison of SM effects on thermal and colloidal stability.

Here, we use literature data, as well as new measurements performed for this paper, to qualitatively compare the effects of SMs on the thermal and colloidal stability of two model proteins: lysozyme and human carbonic anhydrase (**hCa**). The resulting changes in *T*_*m*_ and *B*_22_ induced by the addition of various SMs are shown in Figure 1.

**Figure 1:**
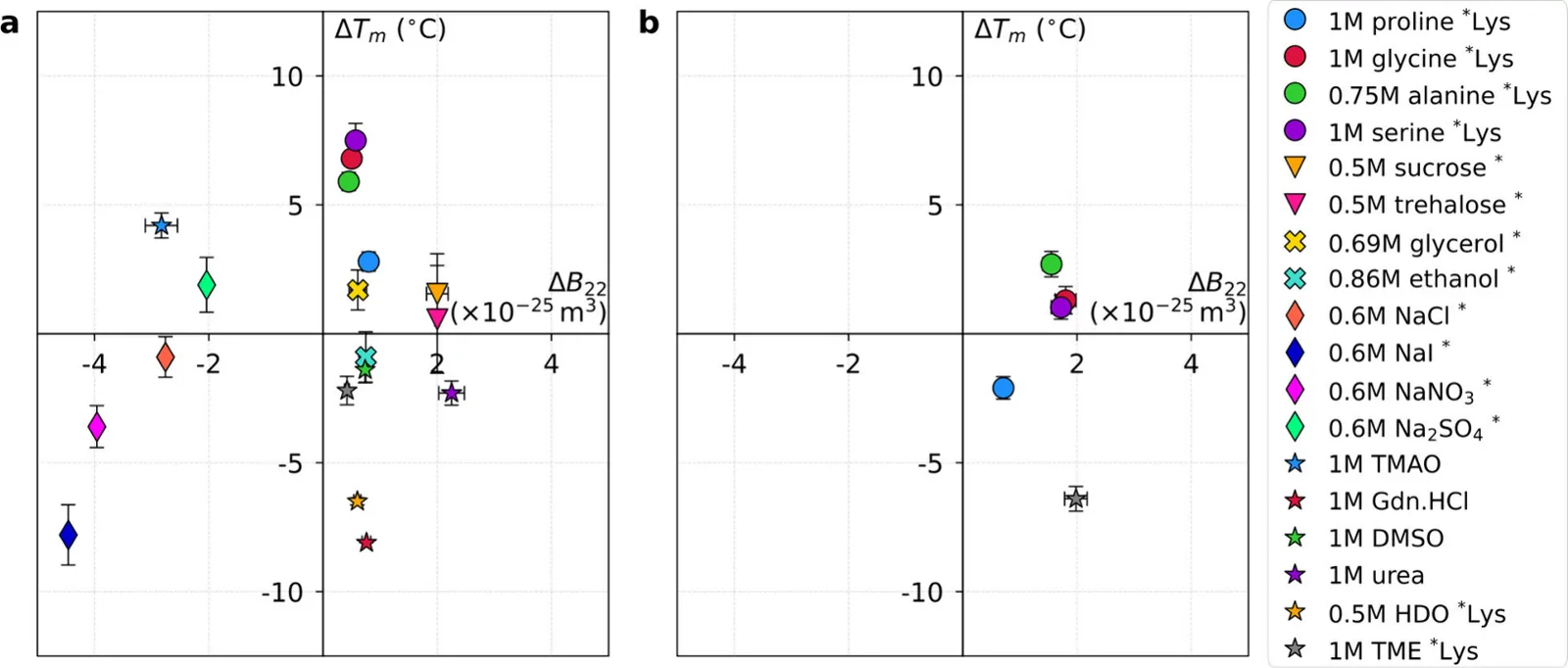
Correlation between the change in thermal stability (*ΔT*_*m*_) and the change in the second virial coefficient (*ΔB*_22_) of (**a**) lysozyme and (**b**) hCa on addition of the SMs at the concentrations indicated in the legend. In the legend, symbols (*) represent *B*_22_ values taken from the literature; (*Lys) marks SMs for which a literature *B*_22_ was available for lysozyme only, the corresponding hCa value being measured in-house. Namely, we used literature *B*_22_ data for: salts (NaCl, NaI, NaNO_3_, Na_2_SO_4_) measured by SIC in 50 mM sodium acetate, pH 4.5;^41^ ethanol and glycerol by SLS in 50 mM NaCl, pH 7;^42^ sucrose and trehalose by SIC in 100 mM sodium acetate, 5% (w/v) NaCl, pH 4.5;^43^ proline, alanine, serine, glycine, TME^8^ and HDO^38^ measured by AUC-SE in 50 mM sodium phosphate, pH 6.9. Where a literature *B*_22_ value at the working concentration (e.g., 1 M) was not measured directly, it was estimated by interpolation of the reported concentration series. Literature values (*B*_2_) originally reported in mass units (mol.mL.g^–2^) were converted in m^3^ units using *B*_22_ = *B*_2_.10^–6^.*M*_*W*_^2^/*N*_*A*_, where 14.3 kDa was used as lysozyme’s molecular weight (*M*_*W*_) and *N*_A_ is the Avogadro number. Data from ref 41 are interpreted as *B*_2_ because comparison with tabulated data indicates that this is the case as opposed to the original incorrect labeling as *b*_2_.

This plot separates SM’s effects into four quadrants: 1) in the upper-right, SMs increase both thermal and colloidal stability; 2) in the lower-right, SMs increase colloidal stability but decrease thermal stability; 3) in the lower-left, SMs decrease both quantities; 4) in the upper-left quadrant, SMs increase thermal stability but decrease colloidal stability.

For lysozyme, all four quadrants are populated (Figure 1a). Most SMs increase *B*_22_, indicating a shift toward more repulsive, or less attractive, PPIs. The amino acids studied here fall in the upper-right quadrant: they increase both *B*_22_ and *T*_*m*_.

In contrast, several other molecules increase *B*_22_ while decreasing *T*_*m*_. 1,1,1-Tris­(hydroxymethyl)­ethane (**TME**), hexanediol, urea, and guanidine hydrochloride (**Gdn·HCl**) fall in the lower-right quadrant. This shows that a molecule can destabilize the folded state of a protein while still making interactions between folded protein molecules more net repulsive. Therefore, thermal destabilization does not necessarily imply colloidal destabilization.

A smaller group of additives decreases *B*_22_ for lysozyme. Several of these are salts, consistent with the idea that electrostatic screening can reduce repulsive PPIs. However, their effects on *T*_*m*_ are not uniform, indicating that the thermal response depends on the ion identity and solution conditions. Trimethylamine N-oxide (**TMAO**) is a particularly notable case because it increases *T*_*m*_ while decreasing *B*_22_. Thus, even a molecule known for stabilizing folded protein structure can promote more attractive native-state PPIs.

For hCA, the molecules we decided to investigate populate only the right side of the plot (Figure 1b). In other words, all tested molecules increase *B*_22_ under the conditions studied. However, their effects on *T*_*m*_ differ. Among the four amino acids tested, alanine has the strongest stabilization effect on *T*_*m*_, while glycine and serine weakly increase *T*_*m*_. In contrast, proline has a weak destabilizing effect on *T*_*m*_ despite increasing *B*_22_. TME decreases *T*_*m*_, as observed for lysozyme. The comparison between lysozyme and hCA highlights the protein-dependent nature of SM’s effects, as observed for proline. This SM has different effects on *T*_*m*_ among proteins under question, yet still produces similar shifts in *B*_22_. These data therefore support the central argument of this Mini-Review: changes in thermal stability and native-state colloidal stability are not generally coupled and should be measured independently.

## Discussion

The data points in the upper-left and bottom-right quadrants of Figure 1 could be explained by the preferential interaction model.^44^ In the case of preferential exclusion, conformationally stabilizing SMs are depleted from the protein surface, resulting in preferential hydration of the protein.^45^ Because exclusion raises the chemical potential of the protein in proportion to its solvent-accessible surface area, equilibria are shifted toward the states that bury the most surface: the native state over the unfolded ensemble, and the associated state over the monomer.^46^ In contrast, preferentially accumulated SMs thermally destabilize proteins by increasing the number of favorable protein–SM interactions in the unfolded state. By the same reasoning, preferential accumulation would disfavor dimerization, as the dimer exposes less surface area than the monomer. However, this interpretation assumes the additivity of interactions, which has not been validated. Strictly speaking, this theory also requires knowledge of the SM’s effects on the folded, oligomerized, and unfolded states, which is difficult to study experimentally. In addition, this model does not explain all observations. For example, it does not explain the effect of proline, which is known to be preferentially excluded and to increase protein solubility. In general, it is a known fact that there is no theory that can explain the relation between *T*_*m*_ and *B*_22_ satisfactory.^47^

We showed that several SMs have the same effect on the colloidal stability of both proteins and gold nanoparticles: TMAO is a destabilizing SM, while urea is a stabilizing SM.^48^ Because gold nanoparticles do not undergo denaturation, the effects of TMAO and urea on colloidal stability cannot be explained only by their effects on protein folding. This further supports the idea that the two concepts should be treated separately before investigating their relationship in protein systems.

Recently, we presented a theoretical approach to explain the stabilizing effect of SMs on the colloidal stability of proteins and, more generally, of colloids. We developed the approach based on the Langmuir-like dependence of protein *B*_22_ on SM concentration.^8^ The framework treats proteins as patchy particles whose net self-interaction is governed by a limited number of attractive surface patches. SMs are assumed to adsorb reversibly onto these sites. The fraction of occupied patches follows a Langmuir adsorption isotherm, determined by the SM concentration, the SM–protein binding constant, and the maximum fraction of the available surface that can be occupied by the SM. Because each blocked patch removes one attractive protein–protein contact, the number of effective patches decreases as SMs bind. This screens the attractive interactions, thereby increasing *B*_22_. Hence, this theoretical framework explains only the stabilizing effect of SMs. So far, only one neutral, nonionic SM has been found to destabilize, namely TMAO, and this cannot yet be explained by the proposed framework. More research is needed to understand the underlying mechanism.

## Conclusion

We think that colloidal and thermal stability should be related in a nontrivial way, and that more research is needed to understand the link between them. Until this understanding is reached, however, it is important to consider them as separate phenomena and to treat the effects of external factors, such as pH, ionic strength, and excipients, on colloidal and thermal stability separately.

## Materials and Methods

### Materials

Hen egg-white lysozyme was purchased from Roche (product no. 10837059001) and used without further purification. Lysozyme stock solutions were prepared directly in buffer and filtered through a 0.2 μm syringe filter before use. Unless otherwise stated, all lysozyme experiments were performed in 50 mM sodium phosphate buffer at pH 6.9. Human carbonic anhydrase (**hCa**) was purchased from Giotto Biotech (Firenze, Italy; product no. G07CA201). The protein stock solution was buffer-exchanged into the final buffer, consisting of 50 mM sodium phosphate and 50 mM NaCl at pH 7.4.

### Thermal Shift Assay

The melting temperature (*T*_*m*_) of both proteins under different solution conditions was determined by a thermal shift assay using an UNcle instrument (Unchained Laboratories, Pleasanton, CA, USA). The final protein concentration was 2 mg/mL in all samples. Each sample was measured in triplicate. Samples were incubated for 180 s, then heated from 25 to 95 °C at 0.3 °C/min. Proteins were excited at 295 nm, and emission spectra were recorded from 240 to 700 nm. The ratio of fluorescence intensities at 350 and 330 nm was plotted as a function of temperature. *T*_*m*_ was defined as the midpoint of the resulting sigmoidal transition. *ΔT*_*m*_ values were calculated as *ΔT*_*m*_ = *T*_*m,SM*_ - *T*_*m,buffer*_ and the average *ΔT*_*m*_ was calculated as the arithmetic mean over the three replicates; the error on *ΔT*_*m*_ was calculated using the error propagation rule.

### *B*_22_ Measurements

*B*_22_ values for protein self-interactions were obtained from sedimentation equilibrium analytical ultracentrifugation (**AUC-SE**), following the procedure described.^8,49^ In a typical AUC-SE experiment, protein samples at 10 mg/mL were loaded into 3 mm path-length AUC cells. Sedimentation–diffusion equilibrium was reached at 20 °C at 36 krpm for lysozyme and 25 krpm for hCa. The densities of the SM solutions were measured with a DMA 4200 M densitimeter (Anton Paar, Graz, Austria). The resulting protein concentration gradients were converted into equation-of-state curves and used to determine *B*_22_. Δ*B*_22_ was calculated as the difference between the *B*_22_ value in the presence of a small molecule and the *B*_22_ value in buffer. For *B*_22_ values reported in the literature,^42,43^ the error on Δ*B*_22_ was calculated using the error propagation rule, except for salts for which no errors have been reported. For in-house AUC measurements, the typical error of this technique, based on the reported data,^8,38^ is 10%. Therefore, we calculated the error bars for each measurement based on this error and added them to Figure 1. Some of the error bars are smaller than the symbol size.
